# Design of Non-Deterministic Quasi-random Nanophotonic Structures Using Fourier Space Representations

**DOI:** 10.1038/s41598-017-04013-z

**Published:** 2017-06-16

**Authors:** Shuangcheng Yu, Chen Wang, Yichi Zhang, Biqin Dong, Zhen Jiang, Xiangfan Chen, Wei Chen, Cheng Sun

**Affiliations:** Department of Mechanical Engineering, Northwestern University, Evanston, IL 60208 USA

## Abstract

Despite their seemingly random appearances in the real space, quasi-random nanophotonic structures exhibit distinct structural correlations and have been widely utilized for effective photon management. However, current design approaches mainly rely on the deterministic representations consisting two-dimensional (2D) discretized patterns in the real space. They fail to capture the inherent non-deterministic characteristic of the quasi-random structures and inevitably result in a large design dimensionality. Here, we report a new design approach that employs the one-dimensional (1D) spectral density function (SDF) as the unique representation of non-deterministic quasi-random structures in the Fourier space with greatly reduced design dimensionality. One 1D SDF representation can be used to generate infinite sets of real space structures in 2D with equally optimized performance, which was further validated experimentally using light-trapping structures in a thin film absorber as a model system. The optimized non-deterministic quasi-random nanostructures improve the broadband absorption by 225% over the unpatterned cell.

## Introduction

Unlike the conventional photonic crystals with periodic structures, quasi-random nanostructures comprise seemingly random material distributions governed by underlying structural correlations. Their quasi-random geometries lead to rich Fourier spectrums that are highly desirable for wide-angle and broadband light control^1, 2^. Such class of nanostructures was discovered in the feathers and scales of multiple lineages of birds and insets for producing non-iridescent colors to fulfill biological functions, such as camouflage, intimidation and communication^2–13^. Taking the inspiration from nature, man-made quasi-random nanostructures have also been developed for various optical applications including energy harvesting^14–18^, light-emitting diodes^19^, random lasing^20, 21^, structural coloration^22, 23^, plasmonic quasi-crystals^24^, transmission and reflection controlling^25–28^.

**Figure 1:**
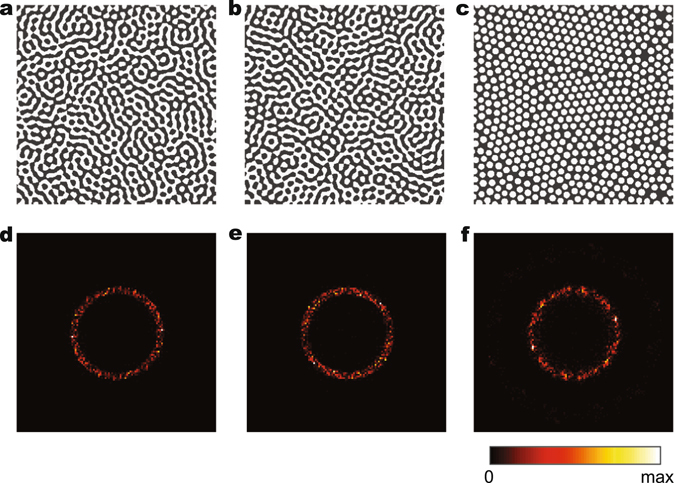
One-to-multiple mapping from Fourier space characterization to real-space geometries of non-deterministic quasi-random structures. (**a**,**b**) Two quasi-random structures with channel-type morphology that can be obtained from spinodal decomposition process (**c**) A disk-type quasi-random structure that can be obtained from nucleation based phase separation process. (**d**–**f**) The Fourier spectra of the structures in (**a**–**c**), respectively.

Apart from their unique characteristics, the design of the quasi-random nanophotonic structures fundamentally differs from the one for their periodic counterparts, mainly due to their inherent non-deterministic nature. To better illustrate this, let us consider several representative cases of the quasi-random structures featuring both channel-type and disk-type morphologies (Fig. 1). Two quasi-random nanostructures with channel-type morphology are shown in Figure 1a and b, which can be obtained via the spinodal decomposition process^9, 29^. On the other hand, Figure 1c shows a disk-type quasi-random structure that can be obtained from nucleation process^30^. Interestingly, despite their drastically different real space appearances, the two-dimensional (2D) Fourier transformations of these structures exhibit very similar ring-shaped patterns as shown in Figure 1d,e. The similarity in the Fourier space not only reveals the underlying structural correlation in common but further suggests their similar characteristics in light scattering or diffraction modes.

However, this observed one-to-multiple mapping from the Fourier space characterization to the real space geometries has been unfortunately overlooked. The current design approaches mainly rely on the deterministic representation of the quasi-random structures in real space^31, 32^. The geometry is first represented using discretized pixels and the material occupations at each pixel are then optimized for the targeting performance. While this real space design approach has seen successes in optimizing periodic photonic structures^31, 33^, it fails to capture the inherent non-deterministic characteristic of the quasi-random structures and inevitably results in a large design dimensionality^31^. Furthermore, in view of the observed one-to-multiple mapping, there are infinite real space patterns that can all satisfy the same design objective by sharing the same type of Fourier spectrum. However, the design based on the real space representation can only provide one specific solution among the infinite choices of quasi-random patterns and thus, lose the genericity of the obtained optimal solution.

On the contrary, the naturally occurring quasi-random nanostructures feature non-deterministic material distributions and are created via extremely scalable and low cost phase separation processes^9^. We recognize that the distribution of the Fourier components in reciprocal space provides a unique structural representation of the non-deterministic quasi- random systems and captures the characteristic of light-matter interaction at the same time. As shown in Figure 1d–f, the concentration of the Fourier components over the narrow range of spatial frequencies in these Fourier spectrums indicates the well-defined correlation length, which has been demonstrated to largely determine the light scattering characteristics of the nanostructures. Therefore, designing the quasi-random structure directly in Fourier space provides a more attractive solution that can simultaneously capture both characteristics of structural correlation in the real space and the light scattering in the reciprocal space.

## Results

In this work, we use the spectral density function (SDF), a one-dimensional (1D) function as the normalized radial average of the square magnitude of the Fourier spectrum, to provide a unique representation of the structure in the reciprocal space. For a two-dimensional quasi-random structure Z(r) with binary value at each location r to represent the material occupation, the SDF *f*(*k*) can be obtained based on the Fourier transformation of Z(r) as Eq. 1:

1 $$\begin{array}{rcl}{F}\{Z({\bf{r}})\} & = & {A}_{{\bf{k}}}\cdot {e}^{i{\varphi }_{{\bf{k}}}},\\ f({\bf{k}}) & = & {({A}_{{\bf{k}}})}^{2}/C,\end{array}$$

where *A*_**k**_ and *ϕ*_**k**_ represent the magnitude and phase information at each **k** of the Fourier transform, and **k** is a frequency vector having the same dimension as Z(**r**). When the quasi-random structure *Z*(**r**) is isotropic, the vector **k** can be reduced to a scalar *k*. Thus, *f*(**k**) becomes a one-dimensional function *f*(*k*) that satisfies $f(k)=\,f(|{\bf{k}}|=k)$, and *C* in Eq. (1) is a normalizing constant that ensures the integral of *f*(*k*) over the considered spatial frequency domain as 1.

The underlying mathematical connection between the real space structural correlation function, i.e. two-point correlation function, and the Fourier spectrum has been well established based on the Winner-Khinchin theorem^34^. Thus, the SDF essentially characterizes the structural correlation information in the reciprocal space. Meanwhile, by describing the distribution of Fourier components over spatial frequency, this characterization method has been utilized for predicting the reflectance spectra of nanostructure in analyzing biological structure color systems^6, 35^. Therefore, the SDF unifies the underlying structural correlation and characterizes the light-matter interaction. By recognizing the axial symmetry in their Fourier spectra, we propose the one-dimensional SDF as a unique representation of the non-deterministic quasi-random nanophotonic systems in two or three dimensions. Therefore, in contrast to the deterministic pixelated real-space structure representation, infinite numbers of the real space quasi-random structures can be generated using statistical reconstruction methods^36, 37^ based on a given SDF structural representation.

**Figure 2:**
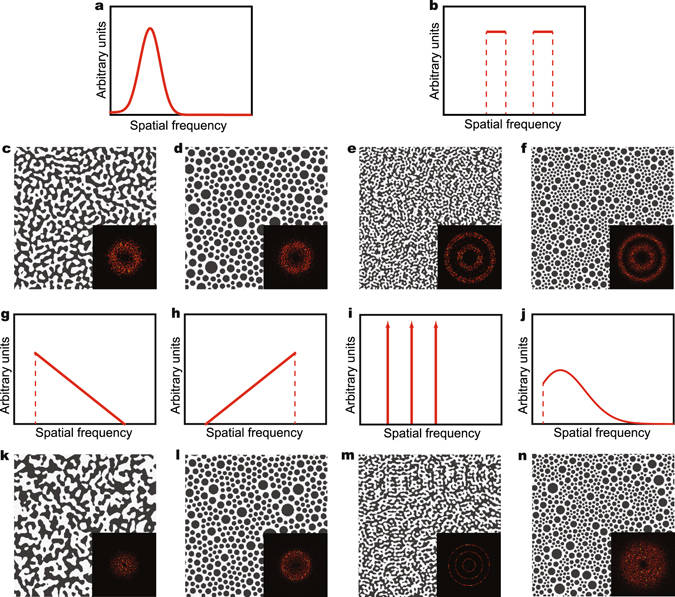
Real-space quasi-random structure reconstruction from SDF. (**a**) An SDF following the Gaussian distribution function. (**b**) An SDF following two-step function. (**c**) A channel-type quasi-random structure reconstructed from (**a**) using Gaussian random field modeling. (**d**) A disk-type quasi-random structure reconstructed from (**a**) using random packing algorithm. (**e**) A channel-type quasi-random structure reconstructed from (**b**) using Gaussian random field modeling. (**f**) A disk-type quasi-random structure reconstructed from (**b**) using random packing algorithm. (**g**) An SDF following a decreasing-linear function. (**h**) An SDF following an increasing linear function. (**i**) An SDF following a triple-delta function. (**j**) An SDF following a truncated Gaussian distribution function. (**k**) A channel-type quasi-random structure reconstructed from (**g**) using the Gaussian random field modeling. (**l**) A disk-type quasi-random structure reconstructed from (**h**) using the random packing algorithm. (**m**) A channel-type quasi-random structure reconstructed from (**i**) using the Gaussian random field modeling. (**n**) A disk-type quasi-random structure reconstructed from (**j**) using the random packing algorithm.

To demonstrate the capability of using SDF for representing and reconstructing quasi-random nanophotonic structures, different forms of SDF are tested as shown in Figure 2. Based on a SDF following a Gaussian distribution shown in Figure 2a, a channel-type and a disk-type quasi-random structure are reconstructed as shown in Figure 2c and d, respectively. Here, we develop two non-iterative reconstruction methods based on SDFs, i.e. the Gaussian random field (GRF) modeling for the channel-type structures and the random packing algorithm (RPA) for the disk-types (see Method Section). Corresponding to the identical SDF, the two structures in Figure 2c and d possess dispersed Fourier spectra as shown in the insets of Figure 2c and d, which implies that their structural correlation spreads over a range of spatial distance. For comparison, a two-step shaped SDF and the two reconstructed structures with different real space morphology types are displayed in Figure 2b,e and f, respectively. The reconstructed structures in Figure 2e and f present a hierarchical geometry with features at two length scales corresponding to the two distinct frequency bands in Fourier space. This interesting feature implies these structures possess structural correlations in two separated spatial ranges. Figure 2g–j display another 4 different SDFs, i.e. the decreasing-linear function in Figure 2g, the increasing-linear function in Figure 2h, the triple-delta function in Figure 2i, and the truncated Gaussian distribution in Figure 2j. Figure 2k–n show the quasi-random structures reconstructed from the SDFs in Figure 2g–j. The distributions of the Fourier components of these structures as shown by the insets of Figure 2k–n correspond to their SDFs. As demonstrated in Figure 2, most SDFs as the non-deterministic representations of the quasi-random structures can be mathematically formulated based on less than 5 governing parameters, which significantly reduces the design dimensionality of such complex systems. Furthermore, since our reconstruction methods are applicable to arbitrary forms of SDF, we can generate the structures mimicking the self-assembled structure from certain bottom-up process by utilizing the corresponding SDF derived in literature^38^.

**Figure 3:**
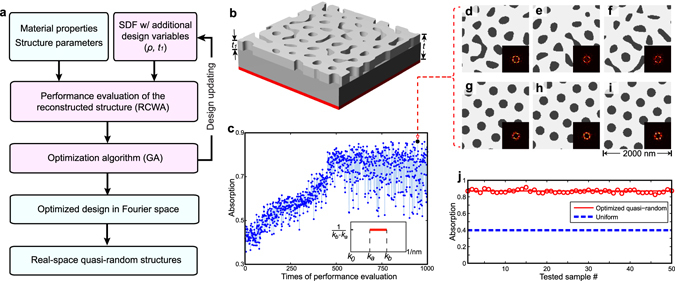
Fourier space design strategy of non-deterministic quasi-random nanophotonic structures. (**a**) The simulation-based design approach for optimizing quasi-random nanophotonic structures in Fourier space based on the structural characterization using SDF. (**b**) The schematic view of the simplified thin-film silicon solar cell model with quasi-random light-trapping structure. The cell is characterized by the thickness of the silicon layer t, and the thickness of scattering layer t_1_. (**c**) Optimization history for designing the quasi-random light-trapping structure in the absorber (t = 600 nm, t_1_ = 100 nm) at λ = 650 nm using genetic algorithm. (**d**–**i**) Six different real-space quasi-random structures reconstructed from the optimal solution, where d-f with the channel-type morphology are reconstructed using Gaussian random field modeling and (**g**–**i**) with the disk-type morphology are reconstructed using random packing algorithm. (**j**) The absorption performance of 50 randomly generated quasi-random light-trapping structures from the optimal solution.

Utilizing SDF for structural representation and reconstruction, we developed a new simulation-based design approach for optimizing quasi-random nanophotonic structures by exploiting the design representation in the Fourier space, as outlined in Figure 3a. Instead of following the conventional approach that tailors the real space structure in 2D, our approach optimizes the 1D SDF *f*(*k*) in the Fourier space as the design representation of the structure with reduced dimensionality. Once the SDF design representation is initialized, the 2D real-space quasi-random structures are reconstructed by GRF or RPA methods. The optical performance of these reconstructed structures are then evaluated using the rigorous couple wave analysis (RCWA) or the Finite-different time-domain (FDTD) method. Based on the evaluation, the SDF parameters are updated using optimization algorithm. Finally, the optimized structures in real space are reconstructed based on the optimized SDF which is obtained from the iterative optimization loop.

Our method is mainly focused on the design of the quasi-random nanophotonic structures that exhibit unique polarization-independent optical characteristic. This is often originated from the lack of the long-range translational ordering in the quasi-random structures, which results in the observed rotational symmetry in the two-dimensional (2D) spectrum in the K-space. Thus, in our method, the one-dimensional (1D) spectral density function (SDF) is used as the unique design representation of the 2D quasi-random photonic structures, which is in clearly contrast to the other K-space based approaches that mainly rely on the use of full 2D Fourier spectrum as the design representations^25, 26, 39^. While the use of 1D SDF as the design representation offers the immediate benefit with significant dimensionality reduction of the design space, more importantly, it further enables the design of non-deterministic quasi-random nanophotonic structures, i.e. the structures of different real-space geometries featuring equivalent Fourier spectrum characteristics with rotational symmetry.

A light-trapping structure in a silicon absorber is employed as a representative example to demonstrate this approach, as shown in Figure 3b. In this simplified model system, the top layer with the quasi-random structure is the light-trapping layer of thickness t_1_, which is directly patterned on the amorphous silicon (a-Si) based absorbing layer with total thickness of t. The bottom silver layer prevents the light escaping on the back side. The light-trapping structure is optimized for maximizing light absorption by balancing the competing processes of light reflection and scattering. The design objective is formulated in Equation 2:

2 $$\mathop{{\rm{\max }}}\limits_{Z(f(k),\rho ,{t}_{1})}:A(Z(f(k),\rho ,{t}_{1}),\lambda ),$$

where Z denotes the quasi-random light-trapping structure depending on *f*(*k*) as the SDF, *ρ* as the material filling ratio and *t*_1_ as the patterning depth, *A* is the total light absorption to be maximized at targeting wavelength *λ*.

Our method does not impose any limitation on the formulation of the SDF and thus, designer has the freedom to formulate the SDF according to desired physics optical properties as the design objectives. For the demonstration of this capability, we choose the step-shaped SDF exhibiting band-pass characteristics in the k-space. The corresponding pass-band is defined within the range from *k*_a_ to *k*_b_, which is shown in the inset of Fig. 3c. The unit of *k*_a_ and *k*_b_ is nm^−1^. Since the material refractive index also significantly influences the absorption in light-trapping process, the material filling ratio *ρ* of the quasi-random nanostructure is simultaneously designed. The thickness of the quasi-random nanostructured layer *t*_1_ is another design variable considered in this work. Comparing with the real space approach that involves thousands or millions of design variables due to the design space discretization, this Fourier space design approach with only four design variables, i.e. (*k*_a_, *k*_b_, *ρ*, and *t*_1_), significantly reduces the design dimensionality, yet still directly captures the key factors that influence the performance.

In this work, the rigorous coupled wave analysis (RCWA)^40, 41^ is adopted to evaluate the light absorption coefficients of the reconstructed structures. RCWA is a Fourier-domain-based algorithm that can solve the scattering problems for both periodic and aperiodic structures. The length of the unit cell for RCWA calculation is set as 2000 nm. We studied the light absorbing characteristic of the superstructures formed by spatially tiling of 1) identical unit cells or 2) different unit cell patterns but originated from the same SDF. Theoretically, they are expected to exhibit the same light absorption. While different algorithms can be adopted for design updating, here we use the genetic algorithm (GA)^33, 42, 43^. Mimicking natural evolution with the underlying idea of survival-of-the-fittest, GA is a stochastic, global search algorithm involving the iterative operation of selection, recombination, and mutation on a population of designs^42, 43^. This simulation-based design loop iterates to search for the optimized design of quasi-random nanophotonic structures in Fourier space. Based on the optimized SDF and other variables, infinite sets of equally optimized structures in real-space can be generated via statistical reconstructions, using methods such as the GRF method or the RPA method.

Figure 3c–j display the optimization process and results of using our Fourier space design approach for a single wavelength *λ* = 650 nm. In this case, the thickness of the quasi-random light-trapping structure is set as 100 nm and the total thickness of a-Si layer *t* is set as 600 nm. Therefore, three design variables are considered in this single wavelength optimization case, i.e. *k*_a_ and *k*_b_ for the step-shape SDF and ρ for the material filling ratio. The optimization history is shown in Figure 3c, in which each dot represents the corresponding absorption coefficient for a given set of design variables (*k*_a_, *k*_b_, and *ρ*). Starting from the randomly generated initial designs with the absorption coefficient around 0.4, the optimization converges to the solution (*k*_a_* = 0.0018 nm^−1^, *k*_b_* = 0.0030 nm^−1^, and ρ* = 78%) with the absorption coefficient larger than 0.85. Statistical representation using SDF enables infinite numbers of optimized quasi-random structures to be generated in real space. Six 2 μm-by-2 μm designs were reconstructed based on the optimal solution denoted by the red triangles in Figure 3c, with the channel-type structures in Figure 3d–f generated using GRF method, and the disk-type structures in Figure 3g–i generated using RPA method, respectively. Despite the different real-space morphologies, all designs possess similar ring-shaped Fourier spectra and achieve the equal optimum performance. For further validation, shown in Figure 3j, 50 real space patterns were generated from the optimized design and evaluated to have an average absorption of 0.88 with less than 4% variation due to the discrete meshing in numerical calculation, thereby achieving a 225% enhancement of the absorption in the unpatterned cell.

**Figure 4:**
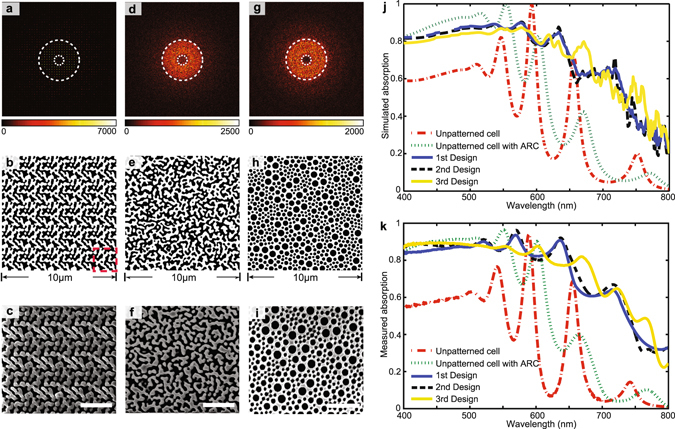
Broadband optimization results. The optimized Fourier spectra, bitmap images, and SEM images of the quasi-random nanostructures generated differently from the same optimized SDF. (**a**–**c**) Quasi-random structure constructed by repeating a single unit cell in the dashed window. (**d**–**f**) Quasi-random structure with channel-type morphology. (**g**–**i**) Quasi-random structure with disk-type morphology. (**j**) Simulated absorption spectra of the three quasi-random nanostructures from the identical optimized SDF, the unpatterned cell, and the unpatterned cell with ARC layer. (**k**) Experimentally measured absorption spectra of the five cells. Scale bars in (**c**,**f**,**i**): 2 µm.

Lastly, we conducted a broadband optimization of the quasi-random light-trapping structures over the visible solar spectrum using our SDF-based design approach. In this case, the total thickness of the a-Si layer *t* is set as 650 nm, and the thickness of the quasi-random structure *t*_1_ is considered as another design variable to demonstrate the strength of our approach. Four design variables (*k*_a_, *k*_b_, *ρ* and *t*_1_) are optimized for the maximal light absorption over the solar spectrum from 400 nm to 800 nm. There are 81 wavelengths considered over the whole wavelength spectrum, and the objective is to maximize the aggregated absorption normalized by the single-path absorption at each wavelength. The optimization converges to the solution (*k*_a_* = 0.0012 nm^−1^, *k*_b_* = 0.0036 nm^−1^, ρ* = 51%, *t*_1_* = 150 nm), which leads to a donut-shape Fourier spectrum as shown in Figure 4a,d,g. This optimized Fourier spectrum overlaps with the area defined by two dashed circles, which denote the range of k-vectors for coupling the incident light to the quasi-guided modes in the silicon film^14, 31^. Three distinct light-trapping designs in real space are realized from the identical optimized SDF. In Figure 4b, the first light-trapping design is generated by repeating one single optimized unit cell, as denoted by the dashed window. A second design with channel-type morphology (Fig. 4e) and a third design with disk-type morphology (Fig. 4h) also are generated by heterogeneous assembly among 25 different unit cells. It is observed here that the quasi-random structures shown in Figure 4e and h possess the rich, continuous Fourier spectra in Figure 4d and g, whereas the periodic structure with quasi-random unit cell in Figure 4b leads to discrete diffraction order in the Fourier spectrum shown in Figure 4a. The gradient of the color bar in Figure 4a has been adjusted to better visualize the discrete points in the Fourier spectrum.

Two reference structures with the same thickness of silicon material are compared with the optimized structures for the light-trapping performance, including unpatterned cells with and without a 70 nm silicon dioxide anti-reflection coating (ARC). The simulated absorption spectra of the three optimized quasi-random structures and two reference cells are plotted in Figure 4j. The unpatterned cell shows overall low absorption except several high peaks due to the Fabry-Perot resonances occurred between the air-silicon interface and the bottom reflector. By adding the anti-reflection coating (ARC), the absorption in high absorbing region of a-Si (400 nm–550 nm) is greatly enhanced, while the Fabry-Perot absorption peaks are suppressed due to the reduced reflection at the air-silicon interface. Furthermore, the cells with the three optimized quasi-random structures from the same SDF exhibit very similar broadband absorption characteristics over the entire spectrum, which is due to the combination of anti-reflection effect and more efficient light scattering. The enhancement in the strong absorbing region of a-Si (400 nm–550 nm) is caused by the reduced refractive index mismatch between the air and the a-Si, which is comparable with the ARC control case. The enhancement in weak absorbing region (550 nm–800 nm) is mainly due to the enhanced light scattering via the design optimization in the K-space. The coupling to the so-called quasi-guided mode results in the enhanced absorption of the incident light. The presence of efficient light scattering also effectively suppress the formation of the Fabry-Perot resonance. All three light-trapping structures with drastically different real-space patterns enhanced the absorption by more than three-fold in the weak absorbing region compared with the two references, achieving an average absorption coefficient of 0.74 over the broad spectrum from 400 nm to 800 nm. All three light-trapping structures with drastically different real-space patterns enhanced the absorption by more than three-fold in the weak absorbing region compared with the two references, achieving an average absorption coefficient of 0.74 over the broad spectrum from 400 nm to 800 nm. The difference between the absorption spectrums of the channel-type structure in Figure 4e and the disk-type structure in Figure 4i is observed. This difference is likely due to the different structure reconstruction methods adopted for the two types of structures. The RPA based reconstruction that requires all the inclusion to have a disk shape has more geometrical constraints than GRF that can generate arbitrary geometries at small scale. Thus, from the same designated SDF, GRF can generate structures more accurately than RPA, resulting in the observed difference between the two absorption spectrums. However, the averaged absorbing performance of these two optimized structures over the targeting broadband is very similar.

The three optimized quasi-random structures were also fabricated on an a-Si substrate and characterized experimentally (see Method Section), as shown in Figure 4c,f and i, respectively. Exhibiting a broadband enhancement, especially over the weak absorption region from 600 nm to 800 nm, the measured spectra of the optimized cells in Figure 4k agreed well with the simulation results. It should be noted that the spectroscopic details found in Figure 4j are caused by the interference of multiple scatterings from the pixelated fine features of the quasi-random pattern in Figure 4b,e,f. In contrast, those fine features such as sharp corners and small dots are diminished from the fabricated samples shown in Figure 4c,f,i, which result in much smoother experimentally measured curve. The quasi-random structure improved the absorption of this a-Si absorber by 230% compared with unpatterned cell without ARC and 147% compared with unpatterned cell with ARC. In this work, the top-down method is employed to fabricate the quasi-random light-trapping structure for conceptual validation. For other bottom-up nanomanufacturing processes^15, 28^, this design approach provides the design freedom to down-select the optimized structures that are compatible with the fabrication platforms. The manufacturing conditions can be further incorporated into the structure design by deriving the formulation of the SDF considering the governing physics of the corresponding bottom-up processes^29, 38^.

## Discussion

In conclusion, we developed a new design approach by adopting the representation in the Fourier space to design the non-deterministic quasi-random nanophotonic structures. The non-deterministic structures in 2D were represented using 1D SDF. This representation in Fourier space not only captures the structural correlation and other key factors that collectively govern the light scattering characteristics of the quasi-random nanophotonic systems, but also significantly reduces the design dimensionality compared with the real space design approaches. We have demonstrated the versatility of the non-iterative method to reconstruct the real space structures with different morphology types based on arbitrary forms of SDF. The Gaussian random field modeling is primarily used to reconstruct quasi-random patterns with “tortuous channel-like” morphology, which can be observed in the processes such as the phase separation by Spinodal decomposition in the polymer blends. In contrast, the random packing algorithm is better suited to reconstruct quasi-random pattern exhibiting particle-like morphology. Thus, RPA can find more applications that relate to nucleation or nanoparticle assembly processes. Following an inverse design strategy, this SDF based approach enables the full exploitation of the potential of quasi-random nanophotonics for high-performance optical devices. It provides the design freedom to down-select the optimized structures that are compatible with the fabrication platforms. The manufacturing conditions can be further incorporated into the structure design by deriving the formulation of the SDF based on the governing physics of bottom-up processes. Readily applicable to various nanophotonic applications, including lasing control, light coupler for LEDs, and structural coloration, this approach can be further extended to solve functional material design problems beyond the optical regime.

## Method

### Disk-type quasi-random nanophotonic structure statistical reconstruction

For the disk-type quasi-random nanophotonic structure shown in Figure 1c, we adopted the random-sphere-packing method to generate the real-space pattern from the given spectral density function. As a statistical reconstruction method, this process achieves the non-overlapping random packing of disks in 2D real-space or spheres in 3D real-space. Many efforts have been made toward the packing algorithm to achieve different geometric characteristics^44–46^. In our work, we developed a new algorithm that packs the disks in 2D space or spheres in 3D space based on its SDF.

Using 2D disk packing as an example, the typical SDF of an equal-size random close disk packing structure in Figure 1c resembles dense hexagonal packings and has apparent periodicity. As a result, its Fourier spectrum its components concentrated on a single ring, i.e. a certain spatial frequency. Through our empirical studies, it is discovered that the radius of the ring in Figure 1f can be estimated based on the radius of disks packed in the structure: *k* = *L*/1.69∙*r*, where *L* is the side length of the structure and *r* is the radius of disks. More complex SDF profiles can be achieved by packing disks with different sizes. It was discovered from our empirical studies that the intensity of SDF *f* (*k*) at a certain frequency *k*_*i*_ is proportional to the total area of the corresponding disk of radius *r*_*i*_, where *k*_*i*_ and *r*_*i*_ satisfy the relationship:

3 $${f}({k}_{i})\propto {N}_{i}{{r}_{i}}^{2},$$

where *N*_*i*_ is the number of disks of radius *r*_*i*_. In addition, when the total areas of disks for each size *r*_*i*_ are equal, the corresponding *f* (*k*_*i*_) are equal. Based on these relationships, the appropriate disk sizes and number of disks can be estimated according to the magnitude of the spectral density over different frequencies and the target material filling ratio. The effectiveness of this packing algorithm is verified by structures with a variety of SDFs in Figure 2.

### Channel-type quasi-random nanophotonic structure statistical reconstruction

To reconstruct the real-space structures with channel-type morphology as shown in Figure 1d for given spectral density functions and material filling ratio, Gaussian random field (GRF) modeling is adopted in this work. A standard GRF, denoted as *Y*(**r**), is a random field with all points jointly following a standard Gaussian distribution. A standard GRF over a *n*-dimensional space is characterized by the field-field correlation function *g*(**r**_1_, **r**_2_) in Equation (4). Defined as the statistical correlation between two points, *g*(**r**_1_, **r**_2_) describes the fluctuation characteristic of the Gaussian random field. In Equation (4), *J* are Bessel functions of the first kind, *f*(*k*) is the spectral density function of a random variable *k*. Therefore, a GRF *Y*(**r**) is constructed for a given spectral density function *f*(*k*) following this relationship. Once the GRF is generated, as shown by Equation (5), the real-space, two-phase, quasi-random structure *Z*(**r**) can be obtained by level-cutting the GRF *Y*(**r**) at *α*, which is determined by the material filling ratio.

4 $$g({{\bf{r}}}_{1},{{\bf{r}}}_{2})={\bf{E}}[Y({{\bf{r}}}_{1})Y({{\bf{r}}}_{2})]={\int }_{0}^{{\rm{\infty }}}\frac{{J}_{(n-2)/2)}(k{\rm{\Delta }}r)}{{(k{\rm{\Delta }}r)}^{(n-2/2)}}\cdot {k}^{n-1}\cdot f(k)dk,{\rm{\Delta }}r=|{{\bf{r}}}_{1}-{{\bf{r}}}_{2}|$$

5 $$Z({\bf{r}})=\{\begin{array}{c}1,\quad Y({\bf{r}})\le \alpha \\ 0,\quad Y({\bf{r}}) > \alpha \end{array}$$

Among the Gaussian random fields, a natural type that has been used to describe the morphology of a thermodynamic system in a phase-separation process is the Gauss-Markov fields. For this type of Gaussian random fields over the 3D space, the Landau free energy is local and a polynomial in (*k* ^2^)^38^. The SDF describing such system is found in literature^38^ as shown in Equation (6), governed by three parameters, *k*_0_, *b*, and *t*, with *C* as the normalization parameter. It has been discussed in the literature^38^ that the length scale of the structural feature in the corresponding channel-type quasi-random system is determined by (**E[** *k* ^2^ **]**)^1/2^ **= **(*b* ^2^ + 2*bk*_*0*_cos*t*)^1/2^ and the minimum feature is determined by the value of *k* as *f*(*k*) → 0. It is noted that the curve approaches to a δ-function with the peak at *k* = b when t → π/2. Other SDF formulations for different thermodynamic systems can be found in literature to describe the morphological characteristic of resultant quasi-random structures.

6 $$f(k)=C\cdot {[({k}^{2}+{k}_{0}^{2})\cdot ({k}^{4}+2{k}^{2}{b}^{2}\cos 2t+{b}^{4})]}^{-\frac{1}{2}}$$

### RCWA absorption evaluation and GA based optimization

In this work, the rigorous coupled wave analysis (RCWA) method is adopted to calculate the light absorption of the real-space quasi-random structures reconstructed from the SDF design representation. RCWA is one of the most commonly used techniques to solve the scattering problem in Fourier space. The length of the unit cell is determined to be 2000 nm in this work. SDF provides a statistical characterization of the structure and different real-space structures (isotropic) reconstructed from the same SDF are statistically equivalent in reciprocal space. The characteristics of the reciprocal space of the structure mostly determine their performance for photon management^31^. Therefore, the light-trapping performance of a quasi-random structure assembled from multiple statistically equivalent unit cells with different real-space geometries is theoretically as same as that of a structure as a periodic arrangement of a same unit cell. In this case, the RCWA calculation while assuming a periodicity boundary condition for either type of the structures returns the correct absorption values, which is validated in Figure 4j and k.

Based on the calculated absorption values, Genetic Algorithm (GA) is used to search the optimal design. The design variables are coded using the binary notation that are regarded as the chromosomes in GA. A population of the chromosomes went through the iterative operation of selection, recombination, and mutation, and converge to the optimal broadband, light trapping structure. Although GAs with different strategies of selection, recombination, and mutation have been developed, the strategy of self-adaptive mutation is used in this work^47^. Here, the designs mutate by adding a random parameter with normally distribution to each design variable. The mutation strength (i.e., standard deviation of the normal distribution) was self-adaptive and varied during the optimization process. The strength increases as the optimization converges. We can observe this self-adaptive control of the mutation in the history plot of main text (Fig. 4a) where larger fluctuations occur at the late stage of the optimization (converged regime, after 186^th^ evaluation) than in the beginning stage (linear regime, before 130^th^ evaluation). In this work, the self-adaptive GA search is performed using the commercial optimization software *iSight* from *Dassault Systemes*.

### Experimental section

The light-trapping structure in thin-film silicon absorber is adopted as the testbed for the SDF based design strategy to optimize quasi-random nanophotonic structures. Fabrication of the amorphous silicon (a-Si) absorber begins with a 200 nm aluminum film deposited on a silicon wafer by an AJA electron beam evaporator. Next, on top of the aluminum reflector, the 600 nm a-Si film was deposited by inductively coupled plasma chemical vapor deposition (ICP-CVD, Oxford Plasmalab 100). In the a-Si with ARC layer, the 70 nm silicon dioxide film was deposited by plasma-enhanced chemical vapor deposition (PECVD, STS LpX CVD). The thicknesses and optical properties of both a-Si and silicon dioxide were characterized by ellipsometer (J. A. Woollam M2000U). The three quasi-random structures in Figure 4 were fabricated by focused ion beam (FEI Helios Nanolab SEM/FIB) milling (30 kV, 28 pA) on the a-Si film with a patterned area of 60 μm by 60 μm. The reflection spectrum for each sample was measured by a grating spectrometer (Andor SR-303i) with wavelength resolution of 0.6 nm combined with a Leica DMI 3000 M microscope. A tungsten lamp was used as a light source covering the spectral range from 400 nm to 800 nm. The reflection coefficient is then calculated by the using the experimentally measured reflection spectrum from an aluminum mirror as the reference. With 500 nm aluminum underneath the a-Si, light transmitted through the sample is negligible. Thus, the absorption spectrum is obtained by subtracting the reflection coefficient from unity.
